# A Digital Diabetes Prevention Program for Hispanic Adolescents (Fit24+): Protocol for a Feasibility Randomized Controlled Trial

**DOI:** 10.2196/75331

**Published:** 2026-04-15

**Authors:** Erica G Soltero, Marbelly Partida, Sandra Mihail, Lizette Villanueva, Callie Lopez, Salma M Musaad, Teresia M O'Connor, Maria J Redondo, Matthew Buman, Debbe Thompson

**Affiliations:** 1 USDA/ARS Children's Nutrition Research Center, Department of Pediatrics, Baylor College of Medicine, 1100 Bates St, Houston, TX, 77030, United States, 1 713-798-7154; 2 Division of Diabetes and Endocrinology, Department of Pediatrics, Baylor College of Medicine, Houston, TX, United States; 3 College of Health Solutions, Arizona State University, Phoenix, AZ, United States

**Keywords:** adolescent health, type 2 diabetes, physical activity, sleep, digital health, health disparities

## Abstract

**Background:**

Hispanic youth are disproportionately impacted by obesity and subsequent type 2 diabetes (T2D) yet remain underrepresented in diabetes prevention research. Digital health interventions hold promise for increasing the accessibility to and engagement in disease prevention programming, particularly among high-risk populations. However, there is a significant gap in the literature regarding digital T2D prevention programs for adolescents or Hispanic youth.

**Objective:**

The objective of this article is to describe the protocol for examining the feasibility of a 12-week digital diabetes prevention program among Hispanic adolescents with obesity.

**Methods:**

Participants (N=40; aged 12-16 years) will be randomized (1:1) to a 12-week intervention group or a control group. Youth in the intervention group will receive access to an e-learning platform with 12 nutrition and wellness video content sessions, a Fitbit Charge 5, and daily SMS text messages grounded in the self-determination theory to promote physical activity. Youth in the standard control group will receive information on diet and physical activity guidelines and guidance on setting behavior change goals. The study findings will focus on the evaluation of feasibility criteria: (1) recruitment of 40 Hispanic adolescents aged 12 to 16 years; (2) retention of 80% of the participants for postassessments; (3) integrity of the study protocol, defined as 70% or higher completion of content sessions and Fitbit wear on 5 days per week or more with response to 80% of SMS text messages when prompted; (4) 10% or lower incidence of technical issues; and (5) 80% or higher satisfaction among participants.

**Results:**

This study was funded in August 2022 and intervention implementation is ongoing. To date, 35 participants have been enrolled. Study findings will be available before December 2026 and will focus on an evaluation of the a priori feasibility criteria on participant recruitment, data collection, integrity of the study protocol, technical issues, and satisfaction. Study findings will also focus on secondary outcomes.

**Conclusions:**

The feasibility and process evaluation data obtained from this study will provide novel insights on the use of digital T2D prevention strategies among Hispanic youth and families and will inform the development of future digital health interventions among high-risk pediatric populations.

## Introduction

Over the past 20 years, Hispanic adolescents have experienced the largest increase in obesity (22.3% to 30.6%) and severe obesity (7.6% to 12.9%) compared with other pediatric and ethnic subgroups [1]. As a result, Hispanic youth have the highest lifetime risk of type 2 diabetes (T2D) and are disproportionately impacted by this disease [2]. When diagnosed with T2D in adulthood, Hispanic adults disproportionately experience diabetes-related complications and hospitalizations, highlighting the downstream consequences of diabetes disparities [3]. Lifestyle interventions focused on improving diet and physical activity (PA) can reduce diabetes risk by 20% among high-risk adults [4]. However, evidence on the impact of lifestyle-based diabetes prevention interventions among Hispanic youth remains limited [5,6].

Among health promotion and disease prevention studies that do focus on Hispanic youth, accessibility remains an issue for reaching and engaging this population [6]. Traditional in-person interventions are typically delivered in 1 geographic location at a set day and time. However, limited transportation, the need for childcare, and conflicting work schedules can limit participation in in-person lifestyle interventions, especially among Hispanic youth and families, who are more likely to be impacted by some of these challenges [7-9]. Innovations in digital health have been recommended for overcoming barriers to accessibility as some digital devices and services allow for the delivery of disease prevention content directly to participants in their home environment [7,10]. By leveraging digital tools, digital health interventions (DHIs) can assist in overcoming geographic and temporal barriers, increasing the reach of these approaches [11]. DHIs that use devices and services already owned and operated by youth, such as smartphones or SMS text messaging, can increase accessibility by being cost-effective [12,13]. Approximately 95% of Hispanic teenagers in the United States report that they own a smartphone, which is comparable to the prevalence of smartphone ownership among non-Hispanic White youth (94%) [14]. Adolescents are the highest users of SMS text message communication, with a median rate of 60 messages sent per day [15], suggesting that the use of these digital tools is a familiar, accessible, and low-cost strategy. More advanced digital solutions such as chatbots can be deployed via SMS text message to provide personalized PA promotion strategies, which has been shown to increase engagement. Chatbots can also provide continuous, on-demand support for behavior change, further increasing the accessibility of health promotion and disease prevention content. However, there is a significant gap in the literature regarding digital T2D prevention programs for adolescents, particularly among Hispanic youth [16,17].

One limitation of existing DHIs is the lack of evidence-based interventions [18]. In addition to established efficacy, interventions that are culturally grounded and family based are essential for increasing the engagement with and effectiveness of prevention efforts among high-risk minority populations [6,19]. This study leverages previous work conducted on the development and testing of a 12-week culturally grounded, family-focused T2D prevention program [20]. This previously tested prevention program is grounded in social cognitive theory and is designed to foster social support and self-efficacy for improving diet and PA through key behavior change techniques, including goal setting and self-monitoring [21]. This 12-week program is traditionally delivered in a clinic or community setting by trained health educators or registered dietitians to cohorts of 5 to 7 families at a time. Feasibility and acceptability for this prevention program were first established in a pilot study (N=15) among Hispanic adolescents (12-16 years) with obesity (BMI ≥95th percentile) [22]. Participants demonstrated significant increases in insulin sensitivity, assessed through an oral glucose tolerance test [22]. The intervention was then refined and rigorously evaluated in an efficacy trial (N=160), which demonstrated significant short-term (12 weeks) improvements in insulin sensitivity and short- and long-term (12 months) improvements in BMI percentile and body fat percentage [20]. Given the demonstrated effectiveness of this evidence-based prevention program, adapting it into a DHI may serve to increase its reach and accessibility among those who need it the most.

To that end, the evidence-based prevention program described above was adapted for remote, digital delivery using a co-design approach, a process that has been previously described in detail [23]. Hispanic adolescents with obesity were engaged using mixed methods strategies, including in-depth interviews, a youth and expert panel, and several interactive activities to guide the development, design, and usability testing of all study components. The aim of this study is to assess the feasibility of a 12-week digital diabetes prevention program among Hispanic adolescents with obesity. We hypothesize that the program will be feasible and acceptable as demonstrated by a priori criteria for recruitment, retention, the integrity of the study protocol, technical issues, and satisfaction. The feasibility and process evaluation data to be obtained from this study will provide novel insights on the use of digital T2D prevention strategies among Hispanic youth and families and will inform the development of future DHIs among high-risk pediatric populations.

## Methods

### Study Design

This 2-armed, pretest-posttest design will include 40 Hispanic adolescents aged 12 to 16 years with obesity (BMI ≥95th percentile). Youth will be randomized (1:1 ratio) to the intervention or control group. Youth in the 12-week intervention group will receive access to an e-learning platform, a Fitbit Charge 5 (Google), and SMS text messages grounded in the self-determination theory. Youth randomized to the control group will receive a 1-page handout on dietary and PA guidelines for adolescents and standard counseling on developing specific, measurable, attainable, relevant, and time-bound (SMART) goals to improve these health behaviors. All participants will undergo data collection at baseline and 3 months after the intervention.

### Participants

We will recruit equal numbers of male and female participants who meet the following specific inclusion criteria: (1) self-identification as Hispanic individuals; (2) age between 12 and 16 years; (3) obesity, defined as a BMI in the 95th percentile or above; and (4) ownership of a smartphone. Participants will be excluded if they (1) take medication or have been diagnosed with a condition that significantly influences their diet or ability to be active, (2) have experienced a recent hospitalization or injury that would impact participation in diet changes or activity, (3) have been diagnosed with T2D, (4) are pregnant, or (5) are currently enrolled in an exercise program or currently using a PA monitoring device. We will recruit 40 youths anticipating 20% attrition, yielding a final sample size of 32. This sample size is adequate for determining feasibility parameters and generating data on preliminary efficacy [24,25].

### Participant Recruitment

Recruitment will be conducted in collaboration with local pediatric clinics, including Texas Children’s Pediatrics, Harris Health pediatric clinics, and smaller pediatric practices across the greater Houston metropolitan area, Texas. These community-based clinics offer preventive services to youth in underserved, predominantly minority communities. Most patients treated in these clinics qualify for Medicaid. The research team will work with each clinic to implement strategies for physicians to refer potentially eligible patients to the study. We will also collaborate with community-based organizations (eg, Houston Public Library and BakerRipley Community Developers) for recruitment. Study information will be disseminated by attending community events (eg, health fairs and back-to-school fairs), hosting health education workshops at partnering organizations, and attending service events such as Houston food bank food drives. The research team includes bilingual and bicultural staff members who share study information and answer questions in English and Spanish to facilitate recruitment.

### Ethical Considerations

The study protocol and all study-related materials have been approved by the Institutional Review Board at Baylor College of Medicine (H-49195). All study-related documents will be available in English and Spanish, with bilingual and bicultural research staff administering consent procedures, conducting data collection, and answering questions. Prior to any study procedures, trained research team members will obtain written parental consent and child assent. Participants will be informed that their participation is voluntary and they are free to withdraw from the study at any time. Participants will also be informed that nonparticipation will not affect any health or medical services they currently receive and that confidentiality will be maintained using unique identification numbers and a password-protected database. Given the use of third-party digital services, business associate agreements, which are legal contracts required under HIPAA (Health Insurance Portability and Accountability Act) to protect data and personal health information, have been exacted with Fitabase (Fitbit data), Mobile Coach (SMS text messaging), and LearnWorlds (nutrition education platform) to ensure that no data are shared outside of these companies or used in any way outside of the study aims. Participants will receive US $140 in compensation for completion of data collection assessments. This study is registered at ClinicalTrials.gov (NCT04953442).

### Procedures

#### Intervention Overview

For the nutrition education component, we will implement a nutrition and wellness curriculum, which is designed to foster self-efficacy and social support to promote behavior changes. Participants will be asked to watch 1 nutrition and wellness content session (25-35 minutes) per week as a family on a day and time that works best for their schedule. Self-efficacy will be fostered through the educational content, which will include nutrition knowledge regarding topics such as macronutrients, portion sizes, and reading nutrition labels. Textbox 1 provides the titles of all 12 sessions. Each session consists of a prerecorded content video available in Spanish or English that includes didactic teaching delivered in a conversational style by 2 bilingual, bicultural research team members. The content videos include interactive features such as quizzes, animations, and additional links to health information on the content topic. To access the content videos, the adolescent participants and all participating family members will use an e-learning platform hosted by LearnWorlds (LearnWorlds Ltd). This platform is a password-protected website and has an accompanying mobile phone app that mirrors the website, allowing participants to access intervention content using a laptop, desktop computer, tablet, or smartphone.

At the start of the intervention, families will receive an intervention toolkit that will include a workbook. Inside the workbook are content session handouts (for the family and for the individual youth), youth-focused activities to reinforce the concepts and behavior change techniques taught in the content sessions, and a section for listing weekly SMART goals for behavior change that will lead to the mastery of skills through goal attainment. Social support will be fostered within families through the family-focused activities and as families are encouraged to support each other toward their SMART behavior goals. Social support will also be fostered as families will receive a monthly phone call from a research team member to answer questions, provide encouragement, and address any technical issues. Families will be asked to work together as a team to make 4 different healthy, culturally appropriate recipes throughout the intervention. To increase the feasibility of making these recipes, the intervention toolkit will also include a few nonperishable food items. For example, one of the recipes is a lentil soup, so each family will receive a can of lentils and a can of beef broth. Brief (1-3 minutes) demonstration videos for each recipe have been developed and are available through the e-learning platform.

Textbox 1. **Nutrition education curriculum topics.**

- Session 1: Getting Started
- Session 2: Health Awareness
- Session 3: Roles & Responsibilities
- Session 4: Keep it Moving
- Session 5: How Sweet Are You?
- Session 6: Champions with Breakfast
- Session 7: Slim the Fat
- Session 8: Fast Food
- Session 9: Snack Attack
- Session 10: Stay Strong
- Session 11: Self-Esteem
- Session 12: Balancing Act

**Figure 1.**
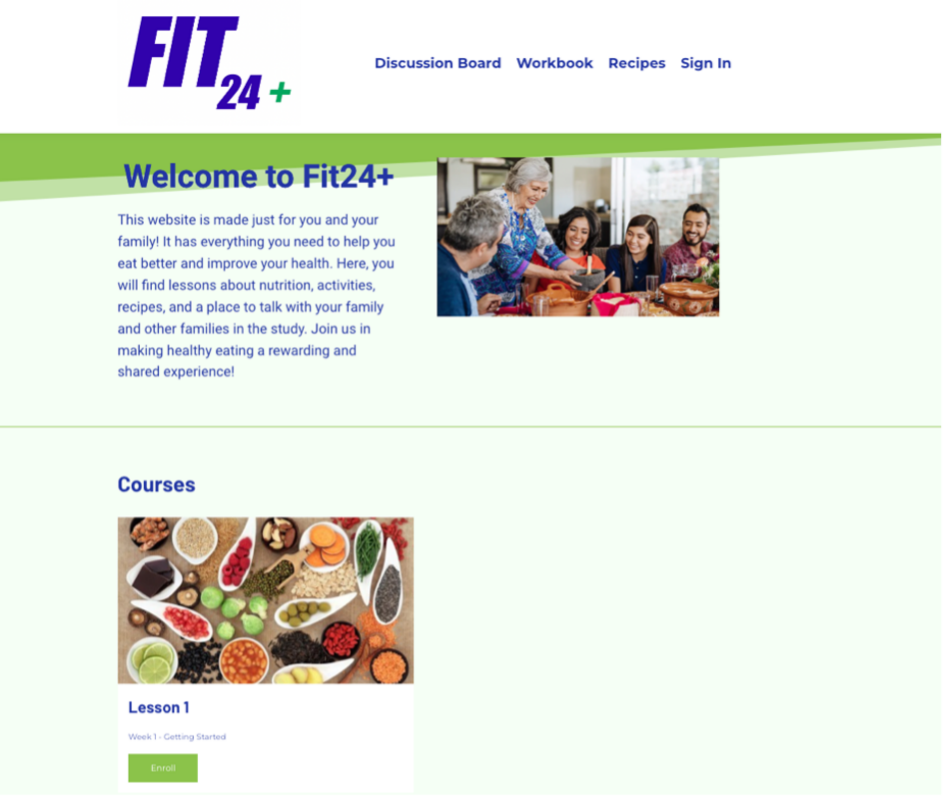
Screenshot of the LearnWorlds e-learning platform.

Additional components have been developed within the e-learning platform to further foster self-efficacy and social support among and within participating families. The platform includes a discussion board, a recipe tab, and a support tab where participants can ask questions about content or report technical issues (Figure 1). Families will be encouraged to use the discussion board to share successes and challenges regarding SMART goals, their experience in trying new recipes, and other tips and strategies for behavior change. The recipe tab will provide families with additional healthy snack, breakfast, lunch, and dinner recipes. The LearnWorlds platform also allows us to connect a secure, institutional Zoom account (Zoom Video Communications) to the platform, which will be used to host monthly live, synchronous sessions between families and the research team. Live sessions will be hosted separately for parents and youth to enable participants to ask questions about the nutrition education content, share their experiences throughout the program, and further share their success and challenges regarding behavior change.

#### PA Component

**Figure 2.**
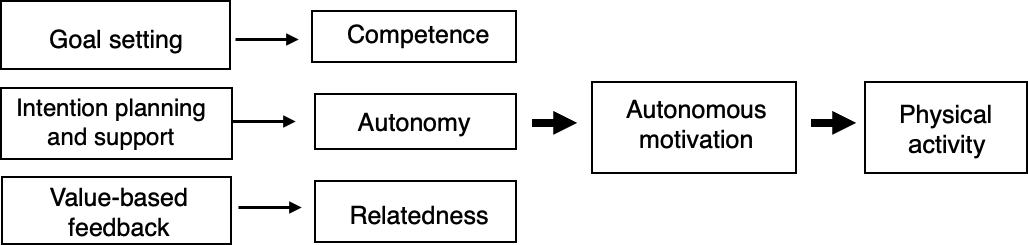
Conceptual map demonstrating how SMS text message components align with self-determination theory constructs (autonomy, competence, and relatedness) to promote autonomous motivation for physical activity.

For the PA component, adolescent participants will receive a Fitbit Charge 5 device. The Fitbit Charge has shown high validity and reliability (intraclass correlation coefficient=0.71-1.00) for assessing steps per day in adolescents [26,27]. Youth will also receive access to Steph, a chatbot that will be deployed via SMS text message. Steph is designed to assist youth in goal setting and intention planning and to provide support for achieving step goals and feedback on goal attainment. The needs support offered by Steph is grounded in the self-determination theory, as shown in Figure 2.

On Mondays, Steph will provide youth with a step goal. In line with the self-determination theory, goal-setting SMS text messages are written in a manner that considers the participants’ autonomy by giving them the choice to accept the step goal that was provided by the research team or allowing them to suggest a goal that is more feasible for them that week. Goals will be adaptive and will increase successively by 10% of the previous week’s performance throughout the 12-week intervention to guide the participants to meet or maintain the current PA recommendation of 12,000 steps per day [28,29]. For the intention planning component, Steph will ask the following questions: (1) “What activities will you do to meet your goal?” (2) “On what day and at what time will you do this activity to meet your goal?” (3) “Why is this goal important to you?” These questions are also designed to promote autonomy by providing control and choice over how the participant will achieve their goal. Steph will also provide accountability by following up with the participant on the days and times they intend to be active. On Tuesdays and Thursdays, Steph will provide competence support by providing the participants with a menu of multiple-choice support options: (1) an evidence-based behavior change technique, (2) tips or strategies for overcoming common barriers to PA, or (3) activity suggestions. On the basis of the participant’s selection, Steph will send a corresponding support message. On Wednesdays, Steph will ask participants to report on their step progress to further encourage self-monitoring toward goal attainment. Asking youth to report on their own step progress as opposed to sending these data to them further engages them in the self-monitoring process [30]. On Saturdays, Steph will send value-based feedback on goal attainment to promote relatedness. These messages will tie goal attainment to commonly held values among Hispanic adolescents, including being healthy and fit and getting good grades [31]. Connecting goal attainment with a self-endorsed value can promote internalization of the behavior so that it becomes integrated into one’s sense of self [32].

Steph is a rule-based chatbot trained on a library of 125 messages that were developed in a previous pilot study conducted by the research team, and she is constrained so that she can only draw from our library of messages [31]. Steph is not capable of 2-way communication and only uses natural language processing to provide youth with PA suggestions when prompted. For example, a participant may ask Steph to recommend a 20-minute aerobic exercise that can be done in the home environment, and Steph will provide the participant with a feasible recommendation.

#### Control Group

Participants randomized to the control group will receive a 1-page informational handout with diet and PA guidelines for adolescents [33]. They will also receive general behavioral counseling information via SMS text message on setting SMART goals to promote healthy diet and activity behaviors.

#### Pre- and Postintervention Follow-Up

All youths, regardless of group assignment, will participate in a baseline preintervention assessment and a 12-week postintervention assessment.

#### Primary Outcomes

The primary outcome of this study is feasibility, which will be evaluated using the a priori criteria outlined in Table 1 [34]. Recruitment and data collection criteria will assess the feasibility of recruiting and retaining high-risk Hispanic youth and families. Process evaluation data on the number of participants screened, enrolled, and assessed will be collected via a REDCap (Research Electronic Data Capture; Vanderbilt University) electronic database. The “integrity of the study protocol” criteria will be used to identify the feasible and acceptable intervention dose [25]. This will be assessed using data extracted from the e-learning platform, including the number of content sessions completed, the frequency of engagement with the e-learning platform measured using the number of log-ins to the website, and the days of Fitbit device wear [35]. Criteria regarding technical issues will assess the feasibility of using an online e-learning platform, a Fitbit device, and an SMS text message–enabled chatbot. Technical issues experienced by the research team and technical issues reported by participants will be logged in REDCap. Satisfaction criteria will assess program acceptability via an exit satisfaction survey. Satisfaction will be further evaluated by conducting exit interviews among all participants randomized to the intervention group. The purpose of these interviews will be to elicit participants’ attitudes, perceptions, and suggestions for improving the program. Interviews will be audio recorded and transcribed, and a thematic content or narrative analysis will be used to identify emergent themes on participant satisfaction [36,37].

**Table 1.** A priori feasibility criteria.

Category	Criteria
Recruitment	Recruiting and enrolling 40 Hispanic adolescents aged 12-16 y
Data collection	Data collection on 100% of the participants at baseline and 80% of the participants at the postintervention time point
Integrity of the study protocol	≥70% completion of content sessions and Fitbit device wear on ≥5 d per wk, with response to 80% of SMS text messages when prompted
Technical issues	≤10% technical issues with the e-learning platform, Fitbit device, and SMS text message transmissions
Satisfaction	≥80% of participants reporting “excellent” to “good” satisfaction with the intervention

#### Secondary Outcomes

The secondary outcomes will include BMI percentile and PA. Height and weight will be measured to the nearest 0.1 cm and 0.1 kg, respectively, and used to calculate sex- and age-adjusted BMI percentile as well as the percentage of participants in the 95th percentile, which is more sensitive to detecting clinical changes in BMI in populations with severe obesity [38-40]. We will assess hemoglobin A_1c_ (HbA_1c_) using a point-of-care analyzer (DCA Analyzer; Abbott Rapid Diagnostics [41]). Given that Fitbit devices provide less accurate measures of PA compared with research-grade devices, a hip-worn accelerometer (ActiGraph wGT3X-BT; Ametris) will be used to assess PA at the pre- and postintervention time points only [42]. Participants will be asked to wear the accelerometer for 7 days and will receive daily SMS text messages to promote compliance in device wear [43,44]. A valid day of wear will be defined as 10 hours or more of wear on 4 days or more, one of which has to be a weekend day [45,46]. Data will be analyzed using Evenson cutoff points in the ActiLife software (Ametris) to report average minutes of moderate to vigorous PA, light PA, and total PA per day [47,48].

#### Tertiary Outcomes

Tertiary outcomes will include autonomous motivation for PA and digital health equity. Autonomous motivation for PA will be assessed using the Autonomous Motivation for Physical Activity scale of the Behavioral Regulation in Exercise Questionnaire [49,50], which has been validated among adolescents [51]. We used the framework for digital health equity developed by Richardson et al [52] to develop a digital health equity survey to assess access to digital technology, digital self-efficacy, and digital literacy among participating adults and youth. This information will be used to assess the degree to which the digital devices and services used in this study were implemented in an equitable manner.

### Data Analyses

#### Power Analysis

A formal power calculation was not used as the primary purpose of this study is to examine feasibility [25,53]. We will oversample and recruit 40 youths with the conservative assumption that 20% will be lost to attrition, leaving a proposed final sample size of 32, which is adequate for estimating feasibility parameters needed to inform a fully powered intervention [24,25]. We will strive to recruit equal numbers of male and female participants.

#### Primary Outcome Analysis

Standard descriptive analyses including mean estimates and their 95% CIs will be performed on process evaluation metrics, including recruitment; data collection; website engagement; device wear; technical issues regarding the e-learning platform, Fitbit devices, and SMS text message transmission; and participant satisfaction as assessed via the exit survey. Descriptive data on process evaluation will be compared with feasibility criteria to determine the overall feasibility of this approach. To reduce bias in the analysis and interpretation, the lead biostatistician is blinded, and the randomization scheme is implemented by a research staff member who is not involved in recruitment or daily interactions with study participants. To assess satisfaction and acceptability of the intervention, in-depth exit interviews will be audio recorded; transcribed verbatim using a professional transcription service; coded by 2 trained, independent coders; and qualitatively analyzed using NVivo (version 9; Lumivero). Thematic content analysis will be used to identify emergent patterns and insights on participant satisfaction and feasibility of the digital health tools used in the intervention [36].

#### Analysis of Secondary Outcomes

Comparisons of BMI percentile, percentage of participants in the 95th percentile, HbA_1c_, and PA between (Mann-Whitney *U* test) and within (pretest to posttest; paired 1-tailed *t* tests and signed rank test) treatment groups will be conducted. While clinically significant changes in secondary and tertiary outcomes are not expected, we will examine changes in the outcomes and clinically meaningful effects to determine the preliminary efficacy of this pilot study [54,55].

## Results

This study is ongoing. Study recruitment began in September 2025 and is projected to conclude in March 2026. At the time of this publication, we have enrolled 35 participants. Study findings will be available before December 2026 and will focus on an evaluation of the feasibility of this study, as well as secondary outcomes (BMI percentile, HbA_1c_, and PA). Study outcomes will be reported using the CONSORT-EHEALTH (Consolidated Standards of Reporting Trials of Electronic and Mobile Health Applications and Online Telehealth) reporting guidelines for DHIs. Study results will be communicated to participating pediatric clinics and community partners through in-person presentations and written reports. The results will also be disseminated through scientific publications, as well as through national and international conferences.

## Discussion

### Anticipated Findings

Only 1 culturally grounded T2D prevention program has been developed and tested among Hispanic youth and families [6]. Despite its efficacy, this in-person program is limited to delivery in 1 location and is time and resource intensive. Because DHIs can leverage commonly owned and operated digital devices and services to deliver intervention content directly to participants, they have the potential for increasing the reach and engagement of evidence-based programs [10]. This paper describes the protocol for examining the feasibility of using an e-learning platform in conjunction with a Fitbit device and chatbot via SMS text messaging for delivering a culturally grounded, evidence-based T2D prevention program among Hispanic youth with obesity and their families.

This study expands upon traditional prevention programs by using digital devices and services to develop a flexible, adaptive intervention that is highly accessible and meets the needs of the focus population. Using an e-learning platform with asynchronous content sessions, a Fitbit device, and a chatbot deployed via SMS text messaging will enable families to access the content on multiple types of digital devices at their convenience, allowing participants to navigate common barriers such as work schedules and other household responsibilities. Use of the chatbot to provide a PA recommendation significantly expands access to behavior change support by providing real-time support that is personalized, contextualized, and accessible when it is needed the most by the participant. Schedule conflicts and lack of program flexibility are consistently named among the most common reasons why parents withdraw their children from traditional disease prevention interventions [56]. Among adults, digital diabetes prevention programs have demonstrated that having highly accessible and flexible content can increase engagement and long-term outcomes [57]. Specific features within the e-learning platform such as recipes and the discussion board were recommended by youth and parents as desired intervention components [23]. Additionally, interacting with the chatbot through action planning was also desired by youth as they recognized the need for assistance in outlining the steps needed to achieve their behavior goals and their need for accountability. Providing a variety of interactive features has also been shown to increase participant engagement and contribute to study outcomes [58,59]. The feasibility and outcome data from this study will provide novel insights on the ability of this digital strategy to impact reach and engagement among Hispanic youth and families.

This study also expands upon traditional prevention programs by focusing on a population impacted by health disparities. Most DHIs are developed and tested on populations with a higher socioeconomic status, and few have focused on high-risk, minority youth [60,61]. This study fills this gap by focusing on Hispanic youth with obesity during the critical life stage of adolescence. The feasibility criteria and additional measures such as the digital health equity survey will provide important information on the user experience, including ease of use, perspectives on functionality, and the accessibility of the digital and intervention components to be used in this study. A recent review of DHIs implemented among adolescents reported that very few studies included any measure of digital health equity [62]. This information is important given that failure to use digital technology in an equitable manner could exacerbate health disparities and potentially contribute to digital disparities [61,62].

### Implications of Anticipated Findings

DHIs that are personalized, adaptive, and accessible have the potential to change the way in which disease prevention programs are delivered in vulnerable communities. According to the US Preventive Services Task Force, improvements in metabolic health requires more than 52 contact hours, which is not feasible in the current health care model [33]. By providing flexible access to nutrition and wellness content through the e-learning platform and the continuous delivery of adaptive, personalized PA content through Fitbit and SMS text messaging, these tools can extend clinical care to meet task force recommendations, possibly leading to greater improvements in metabolic outcomes [63]. Advancements in clinical informatics have demonstrated that data from e-learning platforms and activity trackers can be integrated into electronic health record systems, thus having the potential for DHIs to be integrated into clinical care [64,65]. If found to be feasible, this is a scalable approach that could have significant clinical utility and could make a significant contribution toward addressing T2D disparities within the Hispanic population.

### Potential Limitations

This study is limited by its focus on Hispanic youth with obesity. However, this focus is warranted given the disproportionate impact of T2D on Hispanic youth. In light of the digital divide, there is potential for some Hispanic youths to be excluded from the study due to lack of smartphone ownership. However, recent national statistics have demonstrated that smartphone ownership among Hispanic adolescents is very high (90%) and is comparable to smartphone ownership among non-Hispanic White youth. We will carefully document the number of participants who are excluded due to lack of access to a smartphone. Because youth randomized to the intervention group receive a Fitbit device, SMS text messages, and an online nutrition education platform, there is potential for attention bias that may impact the study effects, which is a limitation. This study is also limited by the small sample size. However, the sample size is appropriate for establishing the feasibility and acceptability of the intervention prior to conducting a fully powered trial that is designed to detect statistically and clinically meaningful changes in health outcomes. Finally, an additional limitation of this study is that it is designed to assess feasibility and, therefore, not powered to identify clinically meaningful changes in weight, BMI, or health behaviors. However, assessing feasibility using metrics that are collected both objectively (eg, recruitment, data collection, technical issues, and device wear) and subjectively (eg, satisfaction) will fill a knowledge gap and inform the development of future DHIs for high-risk adolescents.

### Conclusions

There is a critical need for T2D prevention research focused on high-risk Hispanic youth and families with obesity. This study will deliver a digital T2D prevention program to address growing T2D disparities among this population using an e-learning platform, a Fitbit activity tracker, and SMS text messages grounded in the self-determination theory. This study will lead to novel insights on the feasibility of using these digital tools and services to deliver disease prevention programming to Hispanic youth and families. If found to be feasible, this study will guide the development and implementation of DHIs among other high-risk pediatric populations.

## Abbreviations

CONSORT-EHEALTH: Consolidated Standards of Reporting Trials of Electronic and Mobile Health Applications and Online Telehealth
DHI: digital health intervention
HbA_1c_: hemoglobin A_1c_
HIPAA: Health Insurance Portability and Accountability Act
PA: physical activity
REDCap: Research Electronic Data Capture
SMART: specific, measurable, attainable, relevant, and time-bound
T2D: type 2 diabetes
